# 15 Fertility in juvenile idiopathic arthritis

**DOI:** 10.1093/rheumatology/keac496.011

**Published:** 2022-10-07

**Authors:** Ahlem Ben Ammou, Safa Rahmouni, Soumaya Boussaid, Houssem Tbini, Samia Jemmali, Sonia Rekik, Khaoula Zouaoui, Hela Sahli, Mohammed Elleuch

**Affiliations:** 1 Department of Rheumatology, La Rabta Hospital, Tunis, Tunisia; 2 University of Tunis El Manar

## Abstract

**Background:**

Juvenile idiopathic arthritis (JIA) is a chronic rheumatic disease seen in children below 16 years of age. However, it may persist into adulthood. The damage caused by the disease can influence the sex life and fertility of adults with long-standing JIA.

**Objectives:**

To study the fertility of patients with JIA.

**Methods:**

We conducted a retrospective study including adults with long-standing JIA according to the International League of Associations for Rheumatology (ILAR) criteria.

A detailed questionnaire was completed for each participant by interviewing them as well as by information obtained from their medical records. We identified among the adult patients, those who are married, their age at marriage, the number of gestation and parity, the age of the first child, miscarriages, and occurrence of menopause.

**Results:**

A total of 29 Patients were enrolled, the M/F sex ratio was 0.71. The mean age was 35.69 ± 11.72 [18–61] years. The mean age of disease onset was 11.10 ± 4.25 [2–16] years. The average disease duration was 24.48 ± 12.76 [1–47] years. The mean BMI was 21.20 ± 4.89 [14.17–27.55] kg/m², 36.9% of patient had a BMI ≥ 25 kg/m². Fourteen patients had hip involvement.

Seven patients (6 women, 1 man) were married (24%). The mean age at marriage was 23.75 ± 7.5 [20–35] years. The mean delay between disease onset and marriage was 10 ± 6.68 [6–20] years. Six patient had children, the mean number of gestation and parity were 2.56 ± 2.88 [0–6] and 2 ± 2.29 [0–6] respectively. Miscarriage was noted in 2 patients. Menopause was observed in 3 patients, the mean age at menopause was 46.67 ± 1.53 [45–48] years.

**Conclusion:**

Most of our patients were not married. This reflects the significant impact of the disease on the social and sexual life of JIA patients at adulthood.

